# Supplementary material for: Cloning and Characterization of Two Novel PR4 Genes from Picea asperata
Source: Int J Mol Sci. 2022 Nov 28;23(23):14906. doi: 10.3390/ijms232314906 (PMC9737788; doi:10.3390/ijms232314906)
Supplement: Supplementary file 1 [file ijms-23-14906-s001.zip › ijms-1918432-supplementary.pdf]

Table S1. Primers used in the study

Primer	Primer sequence (5′–3′)*
PaPR4-a-F	ATGATTACGCCAAGCTTGC
PaPR4-a-R	TTAACAACCCACGAATTGATAGTC
PaPR4-b-F	ATGGCTTCCAAAGTTGCAG
PaPR4-b-R	TTAACAATCAACAAAGGTGTATGTAGT
PaPR4-a-Fq	AGGAGTTGCGATAATGGCCG
PaPR4-a-Rq	CTCACATTGGACGCTTGCTG
PaPR4-b-Fq	TCCGCCAATAATTACGCCCT
PaPR4-b-Rq	CACTTAAGCACTTCCCGCAC
EF-F	AACTGGAGAAGGAACCCAAG
EF-R	AACGACCCAATGGAGGATAC
TIF-F	GGTCTTTCCCCTCATCAA
TIF-R	GAGGATGGTTTTGTTAGCC
EcoRI-PaPR4-a-F	AGGCCATGGCTGATATCGGATCCGAATTCCAGCAAGCGTCCAATGTGA
EcoRI-PaPR4-a-R	CGCAAGCTTGTCGACGGAGCTCGAATTCTTAACAACCCACGAATTGATAGTC
EcoRI-PaPR4-b-F	AGGCCATGGCTGATATCGGATCCGAATTCCAGACGCAGAGCAATACAT
EcoRI-PaPR4-b-R	CGCAAGCTTGTCGACGGAGCTCGAATTCTTAACAATCAACAAAGGTGTATGTAGT
T7-F	TAATACGACTCACTATAGG
T7-R	GCTAGTTATTGCTCAGCG
EGFP-PaPR4-a-F	GTCCCGGGGCGGTACCCGGGATCCATGATTACGCCAAGCTTGC
EGFP-PaPR4-a-R	GGCGCGCCGGGCCCTCTAGAGGATCCTTAACAACCCACGAATTGATAGTC
EGFP-PaPR4-b-F	GTCCCGGGGCGGTACCCGGGATCCATGGCTTCCAAAGTTGCAG
EGFP-PaPR4-b-R	GGCGCGCCGGGCCCTCTAGAGGATCCTTAACAATCAACAAAGGTGTATGTAGT
BamHI-yz-F	ATCACTCTCGGCATGGAC
BamHI-yz-R	GATTCTGGTGTGTGCGCA
OE-PaPR4-a-F	CGAGAATTCGAGCTCGGTACCCGGGATGATTACGCCAAGCTTGC
OE-PaPR4-a-R	AGGTCGACTCTAGAGGATCCCCGGGTTAACAACCCACGAATTGATAGTC
OE-PaPR4-b-F	CGAGAATTCGAGCTCGGTACCCGGGATGGCTTCCAAAGTTGCAG
OE-PaPR4-b-R	AGGTCGACTCTAGAGGATCCCCGGGTTAACAATCAACAAAGGTGTATGTAGT
35S-F	GACGCACAATCCCACTATCC
NOST-R	AGACCGGCAACAGGATTCAATC
PP2A-F	GACCCTGATGTTGATGTTCGCT
PP2A-R	GAGGGATTTGAAGAGAGATTTC
F-BOX-F	GGCACTCACAAACGTCTATTTC
F-BOX-R	ACCTGGGAGGCATCCTGCTTAT

* The restriction sites for vector construction are underlined.

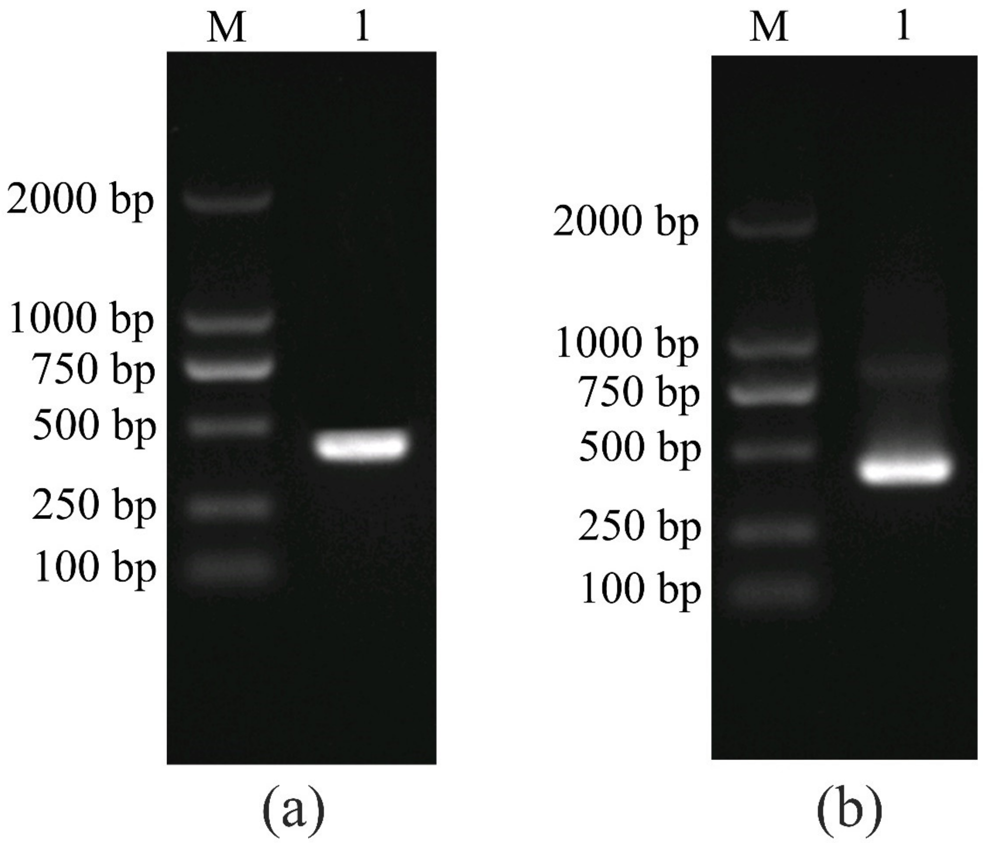

Figure S1. Electrophoretogram of target fragment clones. M: DL2000 DNA marker; (**a**): PaPR4-a target segment; (**b**): PaPR4-b target segment.

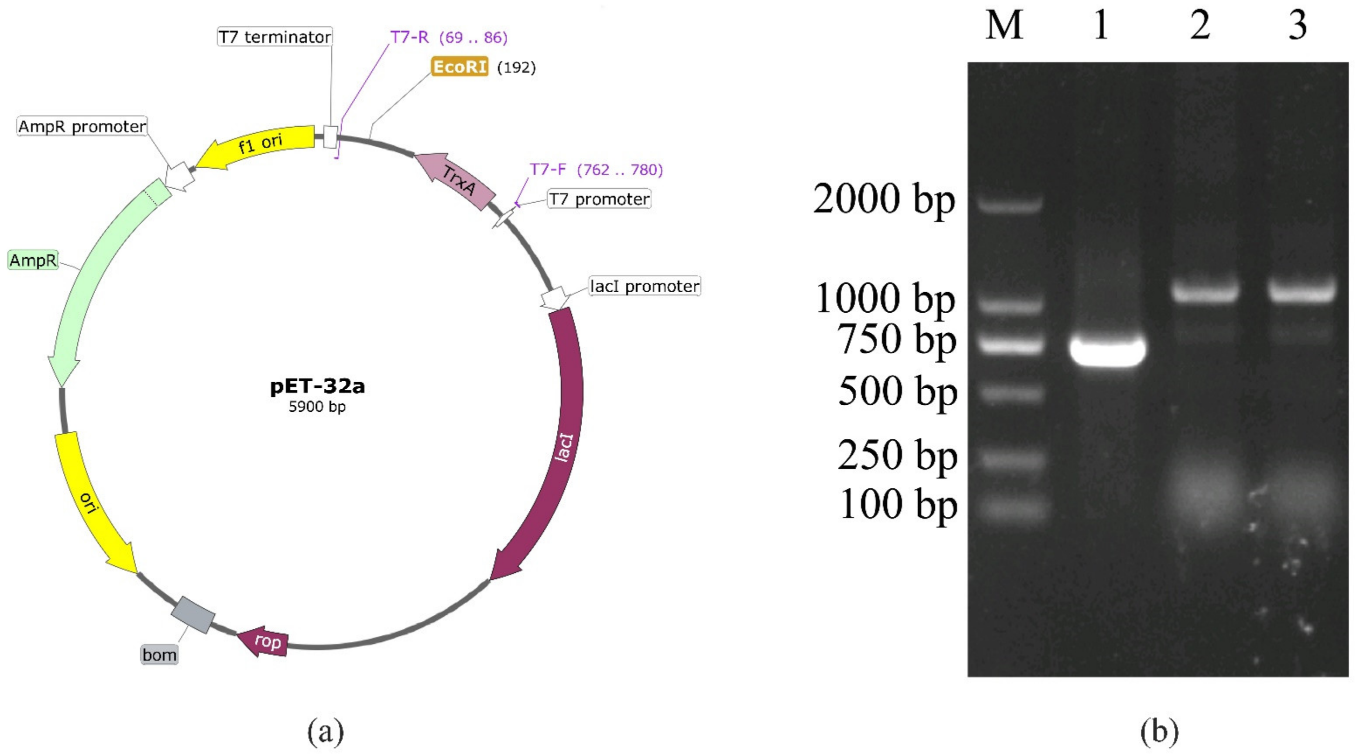

Figure S2. Plasmid map of pET-32a and the results of identification studies. (**a**): Plasmid map of pET-32a; (**b**): M: DL2000 DNA marker; lane 1: PCR amplification product of the empty pET-32a vector; lane 2: PCR amplification product of the pET-32a and PaPR4-a recombinant plasmids; lane 3: PCR amplification product of the pET-32a and PaPR4-b recombinant plasmids.

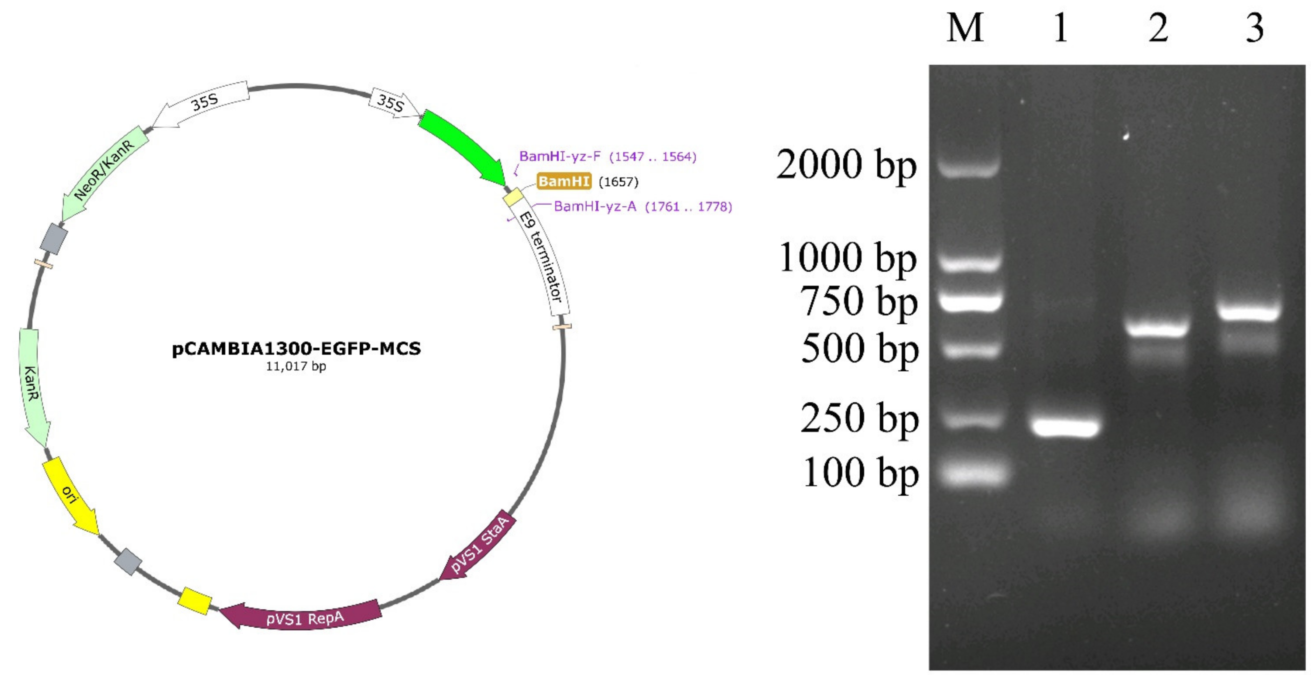

(a) (b)

Figure S3. Plasmid map of pCAMBIA1300-EGFP-MCS and the results of identification studies. (**a**): Plasmid map of pCAMBIA1300-EGFP-MCS; (**b**): M: DL2000 DNA marker; lane 1: PCR amplification product of the empty pCAMBIA1300-EGFP-MCS vector; lane 2: PCR amplification product of the EGFP-PaPR4-a recombinant plasmid; lane 3: PCR amplification product of the EGFP-PaPR4-b recombinant plasmid.

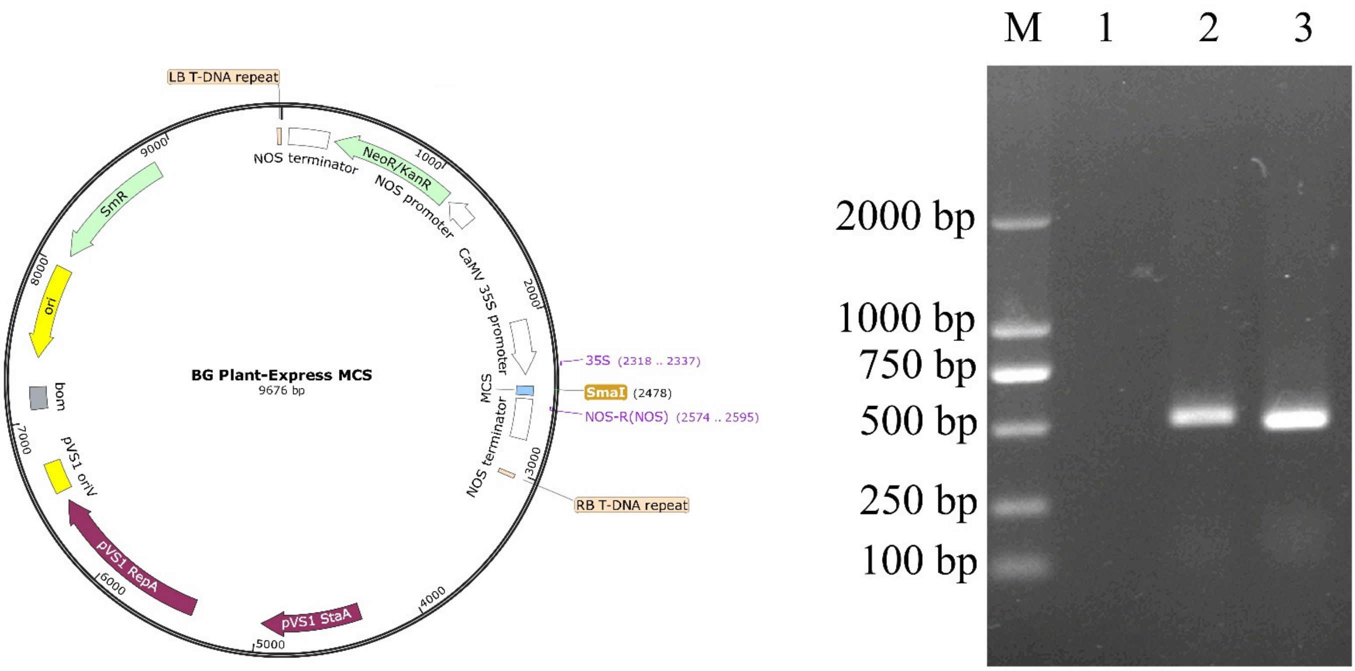

(a) (b)

Figure S4. Plasmid map of BG Plant-Express MCS and the results of identification of transgenic tobacco plants studies. (**a**): Plasmid map of BG Plant-Express MCS; (**b**): M: DL2000 DNA marker; lane 1: PCR amplification product of wild type tobacco; lane 2: PCR amplification product of the PaPR4-a transgenic tobacco; lane 3: PCR amplification product of the PaPR4-b transgenic tobacco.

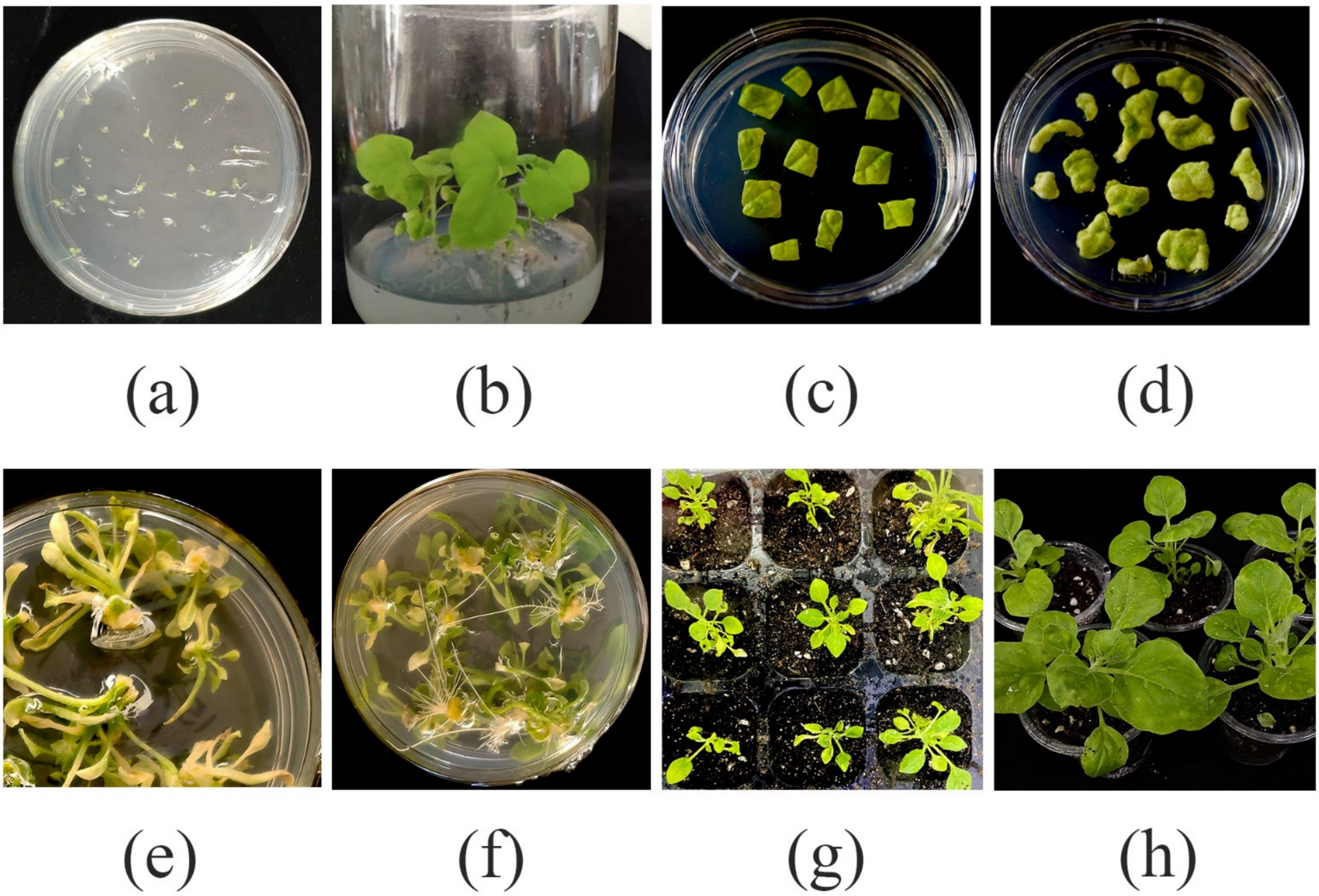

Figure S5. Genetic transformation of *Nicotiana benthamiana*. (**a**): sterilization and seeding of seeds; (**b**): preculture of seeds; (**c**): cutting of explants; (**d**): callus; (**e**): differentiation of buds; (**f**): differentiation of rooting; (**g**): acclimatization; (**h**): after transplanting.
